# Antiviral Activity of Electrospun Polyamide Ultrathin Fibers Against SARS-CoV-2 Variant

**DOI:** 10.1021/acsomega.4c07962

**Published:** 2025-01-21

**Authors:** Moisés V. Santana, Gustavo F. Sousa, Millena C. S. Silva, Lays Cordeiro Guimaraes, Leonardo Camilo de Oliveira, Pedro H. D. M. Prazeres, André S. A. Furtado, Leila S. S. M. Magalhães, Thiago Domingues Stocco, Bartolomeu C. Viana, Ramon Raudel Peña-Garcia, Fernanda Roberta Marciano, Bianca de Sousa Leal, Rosimeire Ferreira dos Santos, João Marcelo de Castro e Souza, Dalton Ditz, Vivian Vasconcelos Costa Litwinski, Mauro Martins Teixeira, Antônio Francisco Machado Pereira, Pedro P. G. Guimarães, Anderson Oliveira Lobo

**Affiliations:** † LIMAV-Interdisciplinary Laboratory for Advanced Materials, UFPI—Federal University of Piaui, Teresina, Piaui 64049-550, Brazil; ‡ Department of Physiology and Biophysics, Institute of Biological Sciences, Federal University of Minas Gerais, Belo Horizonte, Minas Gerais 31270-901, Brazil; § Department of Biochemistry and Immunology, Federal University of Minas Gerais, Belo Horizonte, Minas, Gerais 31270-901, Brazil; ∥ Bioengineering Program, Technological and Scientific Institute, Brasil University, São Paulo, São Paulo 08230-030, Brazil; ⊥ Department of Physics, UFPI—Federal University of Piaui, Teresina, Piaui 64049-550, Brazil; # Academic Unit of Cabo de Santo Agostinho, Federal Rural University of Pernambuco, Cabo de Santo Agostinho, Pernambuco 52171-900, Brazil; ¶ Biochemistry and Pharmacology Department, Health Sciences Center, UFPI−Federal University of Piauí, Teresina, Piaui 64049-550, Brazil; ∇ Department of Cellular Biology, Institute of Biological Sciences, Federal University of Minas Gerais, Belo Horizonte, Minas Gerais 31270-901, Brazil; ○ Nursing Health Sciences Center, UFPI−Federal University of Piauí, Teresina, Piaui 64049-550, Brazil

## Abstract

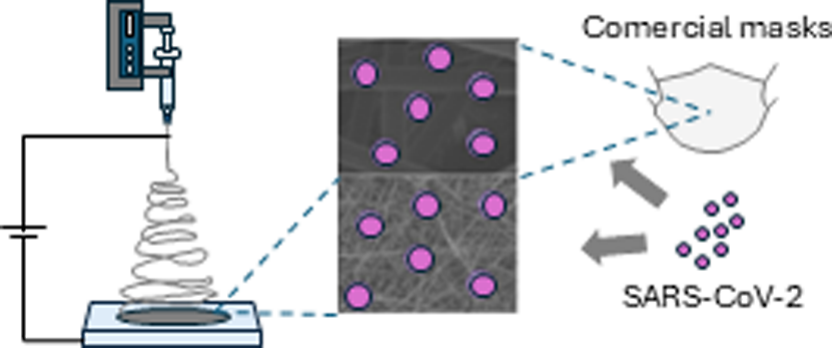

The development of new strategies to produce nanomaterials that can be used as personal protective equipment with antiviral activity and low toxicity is crucial. Electrospun ultrathin fibers have attracted considerable attention due to their potential for biomedical applications, including antiviral activity. Herein, we electrospun different grades of commercially available polyamide to produce ultrathin fibers and investigate their antiviral activity against SARS-CoV-2 Gamma lineage (P.1). We evaluated the morphology, chemical composition, and mechanical properties of the ultrathin fibers. We also investigated the in vitro cytotoxicity, hemolytic activity, and antiviral activity against SARS-CoV-2 Gamma lineage (P.1) of the developed ultrathin fibers. The ultrathin fibers had the following diameters and elastic moduli: (i) unmodified crude ultrathin polyamide (PAP) 0.59 μm and 3 MPa, (ii) polyamide Biotech (PAAM) 0.74 μm and 2.2 MPa, and (iii) Amni Virus-Bac OFF polyamide (PAVB) 0.69 μm and 1.06 MPa. The ultrathin PAP fibers showed increased antiviral activity compared to the other ultrathin fibers (PAAM and PAVB). None of the electrospun fibers showed cytotoxicity at the lowest concentration (12.5%). Additionally, hemolysis tests demonstrated a nonhemolytic profile for all fiber groups, reinforcing their biocompatibility and suitability for biomedical applications. The antiviral properties of the electrospun ultrathin PAP fibers, combined with their noncytotoxic and nonhemolytic characteristics, highlight their potential to be used as personal protection against SARS-CoV-2.

## 1 Introduction

The COVID-19 pandemic has shown that biotechnology is essential for the development of advanced techniques to combat the spread of the SARS-CoV-2 virus. Coronavirus disease 2019 (COVID-19) was a pandemic caused by severe acute respiratory syndrome coronavirus 2 (SARS-CoV-2) and it is also considered a worldwide public health problem. The first symptoms appear after 2–14 days after, ther contact^1^ and the virus is transmitted in aerosol form able to remain for many hours in ambient.^2^ Considering the high number of infected daily in the world, prevention using personal protection device (PPD) is still one of the most important strategies to decrease the spread of the disease. Among these PPD, surgical or textile masks are considered most popular in both hospitals and the streets.^3,4^ During the pandemic most critical crisis with many patients demanding hospitalizations and health care providers working nonstop, the availability of PPD was recurrently at risk owing to of supply shortness, making it extremely necessary to study and manufacture more efficient devices.^5−7^

Even in the postpandemic era, we are suffering from the socio-economic impact and are furthermore vulnerable to new pandemic times of virus spread or even SARS-CoV-2 variables at any time. Thus, are emergent advanced techniques for the production of PPD such as nanofilters and masks to form a physical protective barrier against the spread of pathogens, especially SARS-CoV-2.^8^ The importance of using masks (or nanomasks) in high-risk environments such as hospitals and clinics or in places with high population density and the risk of respiratory disease outbreaks should be emphasized. Bioengineering and biotechnology have proven to be eminent alternative synergies after a devastating pandemic that we have recently experienced with Covid-19.^3,9^ As a result, new physical and biological protection technologies have emerged, such as masks with antiviral and bactericidal properties.^10,11^ The use of masks in enclosed spaces and public transportation is still recommended to prevent the spread of viruses and bacteria in the air.^12^ They can be an important and simple physical barrier to drastically reduce the spread of pathogens like the SARS-CoV-2 virus, by about 95% for particles of size 0.1–0.3 μm,^13,14^ the causative agent of COVID-19, is one of the most aggressive, as demonstrated during the last pandemic.^14^ During the Covid-19 pandemic, the use of masks became essential to contain the spread of the virus.^12^ With the introduction of polymer nanofibers, the masks can provide additional protection and improved functionality.^15^ Polymer nanofibers can filter very small particles such as viruses and bacteria due to their small size and large surface area.^11,15^ As a result, they can effectively trap pathogens and prevent the transmission of diseases.^8^

In addition, polymeric nanofibers can be incorporated into masks to create a breathable, lightweight and comfortable physical barrier for the user. They can also be treated with antimicrobial substances to further increase protection against microorganisms.^16,17^ At the same time, they could replace used commercial masks as they favor the reuse and durability of masks, thus contributing to environmental sustainability by reducing the waste generated by disposable masks.^18^ Therefore, polymeric nanofibers represent a promising alternative to conventional nanomaterials, maintaining the importance of masks as an indispensable public health tool in the postpandemic world.^8,11,19,20^ The production of materials, combined with bioengineering, is making a highly advances, including the production of ultrathin polymeric fibers based on the challenges encountered, resulting in a material capable of meeting many necessity.^21^ Electrospinning surges as an easier, reproducible and scalable technique to produce ultrathin fibers using a simple physical method evolving difference potential and electric field using a high voltage source, a metallic needle and a collector.^22,23^

Synthetic polymers stand out in the production of fibers by electrospinning, among them, polyamides (naylon) due to their thermal stability, durability, and mechanical resistance. The classification of polyamides occurs according to the type of monomers and can be linear aliphatic homopolyamides when created through a type of monomer or linear aliphatic heteropolyamide if created from a polycondension with more than one monomer.^21,24^ Polyamide is therefore frequently used in the manufacture of filter materials. Compared to other biopolymers (such as cellulose, chitosan and alginate^16,25−29^), polyamide offers high filtration efficiency, durability, elasticity, longevity and abrasion resistance. All these properties are related to its molecular structure. By being able to customize the porosity of the material, it can be manufactured to have efficient filtering properties and retain microscopic particles such as viruses and bacteria.^8,15,30^ Integrity, comfort and adaptability are further benefits, as polyamide materials can be designed to be lightweight, soft to the touch and flexible, making it comfortable for the user to wear the mask for extended periods of time.^11,14,17^ In addition, polyamide offers availability and cost advantages as it is widely produced and competitively priced in the market, which can make masks and filters made from this material more affordable compared to more expensive or less common biopolymers.^15,31^

Accordingly, ultrathin polymeric fibers produced by electrospinning emerge as an alternative to the production of fibrous membranes with antiviral activity that can be used as personal protection device (PPD).^32^ Surface modification of ultrathin fibers using physical and chemical methods to modulate hydrophilicity and hydrophobicity is extremely useful to optimize the antiviral activity of these ultrathin fibers.^33^ The contribution of bioengineering is relevant for the development of new and highly effective Materials and Methods to understand pathologies, for the development of smart PPDs and in vitro models that mimic the complex human physiological system.^9,34^ For example, to produce PPE for the prevention of respiratory diseases such as coronavirus disease 2019 (COVID-19). Here, we electrospun polyamide (Naylon) ultrathin fibers using a scalable electrospinning method and evaluated their antiviral activity against SARS-CoV-2 Gamma lineage (P.1). Our best-developed electrospun PPD showed no cytotoxicity, superior antiviral activity, had a diameter of 0.59 μm, regular morphology and porosity, hydrophobic behavior (131 °C), and superior mechanical properties compared to commercial masks that used the same material but with microscopic fiber diameters. We hypothesized that surface modification of the ultrathin fibers would improve their antiviral activity, suggesting that the electrospun polyamide is an interesting strategy to obtain a nano-PPD.

## 2 Materials and Methods

### 2.1 Ultrathin Fibers Preparation

Raw polyamide (PAP) (Naylon), polyamide Biotech (PAAM) and Amni Virus-Bac OFF (PAVB) (all gently supplied from Rhodia company, Sao Paulo, Brazil) were dissolved separately in 5 mL of 1,1,1,3,3,3-hexafluoro-2-propanol (HFIP), Oakwood Products, Inc., Estill, SC, United States (22 wt %) and stirred for 1 h in closed system. The polyamide used is nylon-6–6, which is used to manufacture masks. Then, each solution was loaded into a glass syringe (BD Yale, Burlington, MA, USA) equipped with a needle (Inbras, 22G, Inowrocław, Poland). The electrospinning process was carried out under controlled ambient (temperature 20 ± 2 °C and humidity 46 ± 5%), following the parameters 13 kV (HSensor High-Voltage supply), distance collector-needle of 12 cm, flow rate of 1 mL/h (KDS 100 model, KD Scientific Inc., Holliston, MA, United States) for 3 h. The electrospun ultrathin fibers were named as PAP, PAVB and PAAM groups.

### 2.2 Morphology Characterization

The electrospun ultrathin fibers (neat) and commercial masks (Descarpack) were characterized morphologically by scanning electron microscopy (Quanta FEG 250, FEI, NETHERLANDS). Produced ultrathin fibers were sputter coated with a thin layer of gold using a sputter-coating system before analysis and observed at 15 kV accelerating voltage, spot of 3.5 and work distance of 10 mm. The mean fiber diameters were measured and calculated from the scanning electron microscopy (SEM) micrographs (*n* = 100 fibers) using ImageJ software.

### 2.3 Attenuated Total Reflectance-Fourier Transform Infrared (ATR-FTIR)

The chemical composition was studied by ATR-FTIR (Fourier transform infrared spectroscopy) (Vertex 70 model, Bruker spectrophotometer, German), in the attenuated total reflectance (ATR) configuration. The spectra were found with 60 scans in the 600–4000 cm^–1^ range. The spectra were plotted and treated using OriginLab software.

### 2.4 Thermal Gravimetric Analysis (TGA)

Thermal gravimetric analysis was performed in an air flow at a heating rate of 10 °C/min (STA 449 F3-JUPITER/NETZSCH, German). The analysis was conducted from ambient temperature to 1000 °C at a heating rate of 10 °C/min under a nitrogen atmosphere.

### 2.5 Mechanical Properties

The elastic modulus, tensile strength and fracture strain of the films with different electrospun polyamides ultrathin fibers (PAP, PAAM and PAVB groups) were measured and compared using a texture analyzer (TA.XT plus, USA). Rectangular sample of the films, specifically cut to have dimensions of 10.00 mm × 30.00 mm, were fixed with the probe provided by instrumentation attached to a 5 kg load cell. Measures were carried out at 25 °C and a strain rate of 5 mm min^–1^ (*N* = 3). The findings were expressed as the mean ± standard deviation. Statistical analysis involved conducting a one-way ANOVA followed by multiple Tukey comparisons as a post-test * indicating significance at *p < 0.5, **p < 0.05 and ***p < 0.01.

### 2.6 Contact Angle Measurements

It was used the static sessile drop method for determine the wettability of fibers surface, where the liquid was dropped onto the flat, static substrate (*N* = 3). The liquid/solid interface was registered by a high-resolution camera (Panasonic Lumix FZ-80K model) and the images were used to calculate the contact angle using ImageJ software.

### 2.7 Cell Viability

#### 2.7.1 Ultrathin Fibers Extract Preparation

The ultrathin fibers extracts were prepared according to ISO 10993-5 (ISO, 2009). In a total of 0.1 g of each ultrathin fibers group (PAAM, PAVB and PAP groups), 1 mL of DMEM (Gibco, Invitrogen) culture medium supplemented with 10% fetal bovine serum (FBS; Gibco) was added and the mixtures were stirred at 37 °C for 24 h. The extract obtained (100%) was sterilized in a 0.22 μm pore size syringe filter (Merck Millipore, MA, USA) and diluted to 50%, 25% and 12.5% in DMEM culture medium.

#### 2.7.2 L929 Cell Culture

The L929 mouse fibroblast cell lines were sourced from the Rio de Janeiro Cell Bank (BCRJ, RJ, Brazil). Upon thawing, the L929 cells were cultured in DMEM medium supplemented with 10% FBS and 1% antibiotic-antimycotic solution (100 U/ml penicillin, 100 μg/mL streptomycin, and 0.25 μg/mL amphotericin B; Sigma-Aldrich). These cultures were maintained in 75 cm^2^ Costar flasks (Corning Inc., Corning, NY, USA) under standard conditions of 37 °C and 5% CO_2_, with media changed every 2 days. Upon reaching 80–90% confluence, cells were harvested using 0.05% trypsin–EDTA solution (Gibco) and subcultured into fresh culture flasks.

#### 2.7.3 Cytotoxicity Measurement

Cytotoxicity was assessed by MTT [3-(4,5)-dimethylthiahiazo(-z-y1)-3,5-diphenytetrazoliumromide] assay.^35^ L929 cells were counted using the Trypan blue (Sigma-Aldrich., St. Louis, MO, USA) exclusion method in a Neubauer chamber. The cells were plated in 96 well flat bottom plates using a multichannel pipet at 2 × 10^3^ cell/well. After 24 h, cells were exposed to ultrathin fibers extracts (100%, 50%, 25% or 12.5%) and liquefied phenol (1.6 mg/mL) as positive control (*N* = 5 samples to each group). After 72 h of exposure, 10 μL of 5 mg/mL MTT (Sigma-Aldrich., St. Louis, MO, USA) was added onto each well and incubated at 37 °C with gently stirring for 4 h. Afterward, cell lysis was carried out using dimethyl sulfoxide (DMSO, Sigma-Aldrich., St. Louis, MO, USA), and the absorbance was measured at 540 nm using the Multiskan GO plate reader (Thermo Fisher Scientific, California, USA). The experimental results from three independent trials were graphed as viability percentage (mean ± standard error of the mean) against the concentration of ultrathin fiber extract. Each condition was tested in triplicate wells, and each experiment was replicated three times.

### 2.8 Antiviral Activity

The virucidal activity against SARS-CoV-2 was measured in strict accordance with ISO 18184 guidelines in a Biosafety Level 3 Laboratory (BSL-3). The SARS-CoV-2 clinical isolate used in this study is variant of concern (VOC) belonging to the Gamma lineage (P.1). The SARS-CoV-2 P.1 was kindly provided by Fundação Ezequiel Dias (FUNED, Belo Horizonte, MG, Brazil). SARS-CoV-2 stock was propagated in Vero cells (ATCC—CCL81). The virus stock was titrated using plaque assay in Vero cells as described previously. The SARS-CoV-2 virus stock was used directly in the virucidal tests. Briefly, SARS-CoV-2 P.1 was inoculated onto various mask textiles. A control virus inoculum was prepared as a reference specimen. After specific contact times of 2 and 5 h, the remaining infectious virus was collected in DMEM-2, resuspended, and titrated in Vero cells using a plaque assay to determine the reduction rate. This rate was calculated by comparing the antiviral product test specimen to the reference specimen using a common logarithm. The SARS-CoV-2 virus stock was used directly in the virucidal tests—Ethics Committee from Hospital Eduardo de Menezes (HEM)–Fundação Hospitalar de Minas Gerais, under CAAE 31462820.3.3001.5124—We confirmed that the guidelines outlined in the Declaration of Helsinki were followed. As well as consent to extraction.

### 2.9 Evaluation of Hemolytic Activity in Sheep Blood Agar

The samples were subjected to the hemolytic activity test in sheep blood agar (Laborclin) using the antibiogram technique on discs, adapted for the evaluation of cytotoxicity. For this purpose, the samples were carefully sectioned into 6 mm discs. They were then applied to the medium. The negative and positive controls were, respectively, prepared by impregnating the discs with 20 μL of the solvent used (sterile saline) and Triton (1000 μg mL^–1^). After application, the media were incubated at 35 ± 2 °C for 24 h. After this period, the plates were inspected for the presence of hemolysis halos (measured in mm).

## 3 Results and Discussions

**Figure 1:**
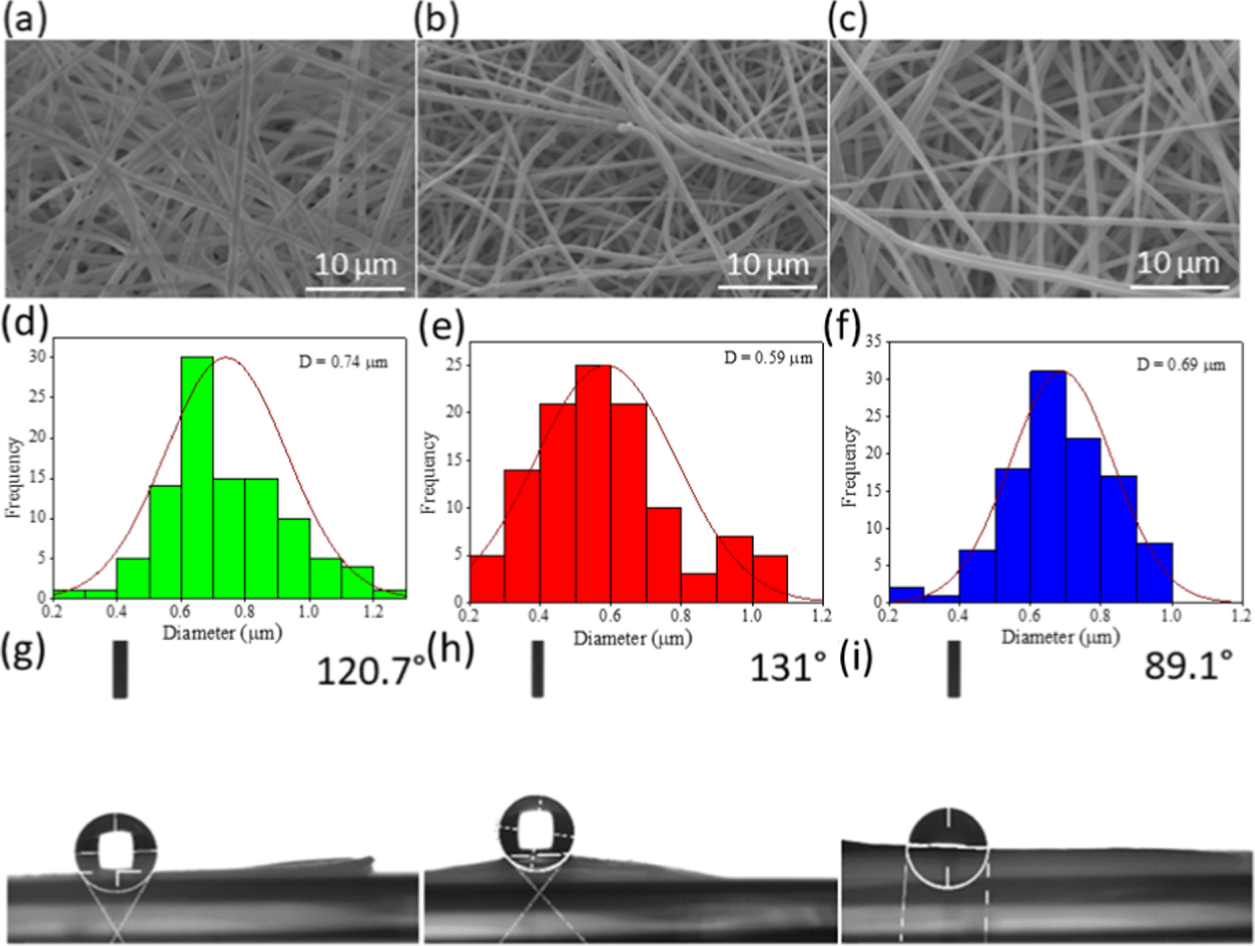
Characterization of the ultrathin fibers. (a–c) SEM micrographs showing the ultrathin fibers morphologies. (d–f) Fiber size distribution. (g–i) Contact angle measurements, the solid–liquid interface. Analyzed groups: (a,d,g) PAAM, (b,e,h) PAP and (c,f,i) PAVB.

In this study, the homogeneous morphology of the electrospun Nylon fibers was a critical factor for their application as face masks. The regular structure of the Nylon fibers, with a consistent diameter of 0.59 μm, ensures uniform filtration efficiency and mechanical strength across the entire membrane (Figure 1a–c). This uniformity is essential for effective particulate capture and user comfort. Electrospinning produces ultrathin fibers with a high surface area-to-volume ratio, which enhances filtration efficiency by providing more surface area for capturing pathogens. The homogeneous morphology of these fibers minimizes variations in pore size, which is crucial for consistent performance in filtering out viruses and bacteria. Irregular fiber structures, on the other hand, can lead to weak points in the membrane, reducing overall effectiveness.

Moreover, the uniformity in fiber morphology contributes to the mechanical robustness of the face masks. Consistent fiber diameter ensures even distribution of stress, preventing localized weaknesses that could lead to tears or failures under mechanical stress. This mechanical integrity is vital for maintaining the protective barrier over prolonged use, especially in demanding environments. Our findings align with previous studies that emphasize the significance of fiber uniformity in electrospun materials for filtration applications. Wang^36^ demonstrated that electrospun Nylon fibers with controlled electrospinning parameters exhibit enhanced filtration properties, making them ideal for mask filters. Similarly, Zeraati^37^ highlighted that regular fiber morphology contributes to the development of efficient filter materials by ensuring consistent pore sizes and filtration performance. The homogeneous morphology of electrospun Nylon fibers not only enhances their filtration efficiency and mechanical strength but also ensures the reliability and durability of face masks made from these materials. This makes electrospun Nylon a promising candidate for developing high-performance personal protective equipment (PPE) for respiratory protection.

The contact angle measurements, depicted in Figure 1g–i, provide crucial insights into the surface properties of the electrospun fibers. The PAVB group exhibited a contact angle of 89.1°, indicative of a hydrophilic behavior. This hydrophilicity is further evidenced by the absorption of the water droplet within 30 s during the test, suggesting enhanced interaction with aqueous environments. In contrast, the PAP and PAAM groups demonstrated contact angles of 131 and 120.7°, respectively, characterizing them as hydrophobic. This hydrophobicity is advantageous for applications such as face masks, where repelling waterborne pathogens and maintaining a dry surface is essential for user comfort and safety.

Polyamide ultrathin fibers typically display hydrophilic behavior with contact angles around 86°.^38,39^ The significantly higher contact angles observed for the PAP and PAAM groups indicate that these fibers have undergone structural modifications to enhance their hydrophobic properties. These modifications could involve or the incorporation of hydrophobic additives, which alter the surface energy and consequently increase the contact angle. The hydrophobic nature of the PAP and PAAM fibers aligns with their potential application in environments where moisture resistance is critical. By preventing the absorption of water, these fibers maintain their integrity and performance over time, reducing the risk of microbial growth and material degradation. This characteristic is particularly beneficial in face masks, where maintaining a dry surface can enhance both filtration efficiency and user comfort.

In summary, the contact angle measurements underscore the distinct surface properties of the electrospun fibers, with the PAP and PAAM groups exhibiting advantageous hydrophobic characteristics due to structural modifications. These properties enhance their suitability for use in protective face masks, providing a balance of mechanical strength, filtration efficiency, and user comfort.

**Figure 2:**
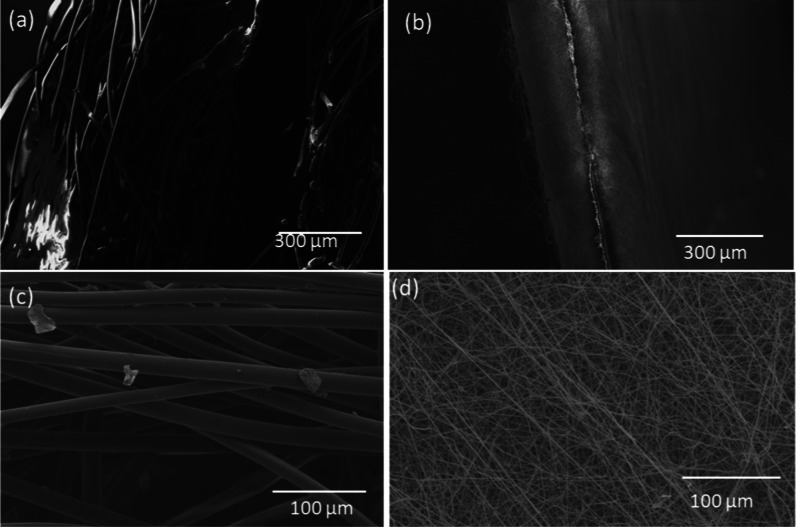
Morphological comparison between commercial surgical masks and electrospun nanofibers. (a,c) A triple layer surgical mask, (b,d) an electrospun produced fiber. (a,b) Side view, (c,d) frontal view.

The SEM images presented in Figure 2 offer a comparative analysis of the fiber morphology and porosity between commercial surgical masks and the electrospun nylon mask. The commercial mask fibers (Figure 2a,c) exhibit noticeable pore defects and larger pore sizes. These structural irregularities can result in greater particle penetration, which may reduce the mask’s overall effectiveness in filtering out viral particles. In contrast, the electrospun nylon fibers (Figure 2b,d) display a more uniform morphology and consistent porosity. This regularity is critical for the development of effective filters, as it ensures a more predictable and reliable filtration performance. The smaller, more uniform pores in the electrospun fibers can trap smaller particles, including viruses, more efficiently compared to the larger and more irregular pores seen in commercial masks.

The development of ultrathin nylon fibers through electrospinning allows for better control over fiber diameter and pore size distribution. This control is beneficial for creating high-performance filters, as consistent fiber morphology and porosity enhance the filtration efficiency while maintaining breathability. The regular structure of the electrospun fibers minimizes weak points that could compromise the mask’s integrity and filtration capabilities. According to Zhang et al. (2021), achieving uniform fiber morphology and porosity is essential for the effectiveness of filtration materials.^40^ The SEM analysis underscores the advantages of electrospinning in producing fibers with superior structural characteristics compared to traditional manufacturing methods used for commercial masks.

In summary, the SEM images in Figure 2 highlight the structural superiority of electrospun nylon fibers over commercial surgical masks. The regular morphology and consistent porosity of the electrospun fibers make them a promising material for developing advanced filtration systems, offering improved particle capture and protection against viral particles.

**Figure 3:**
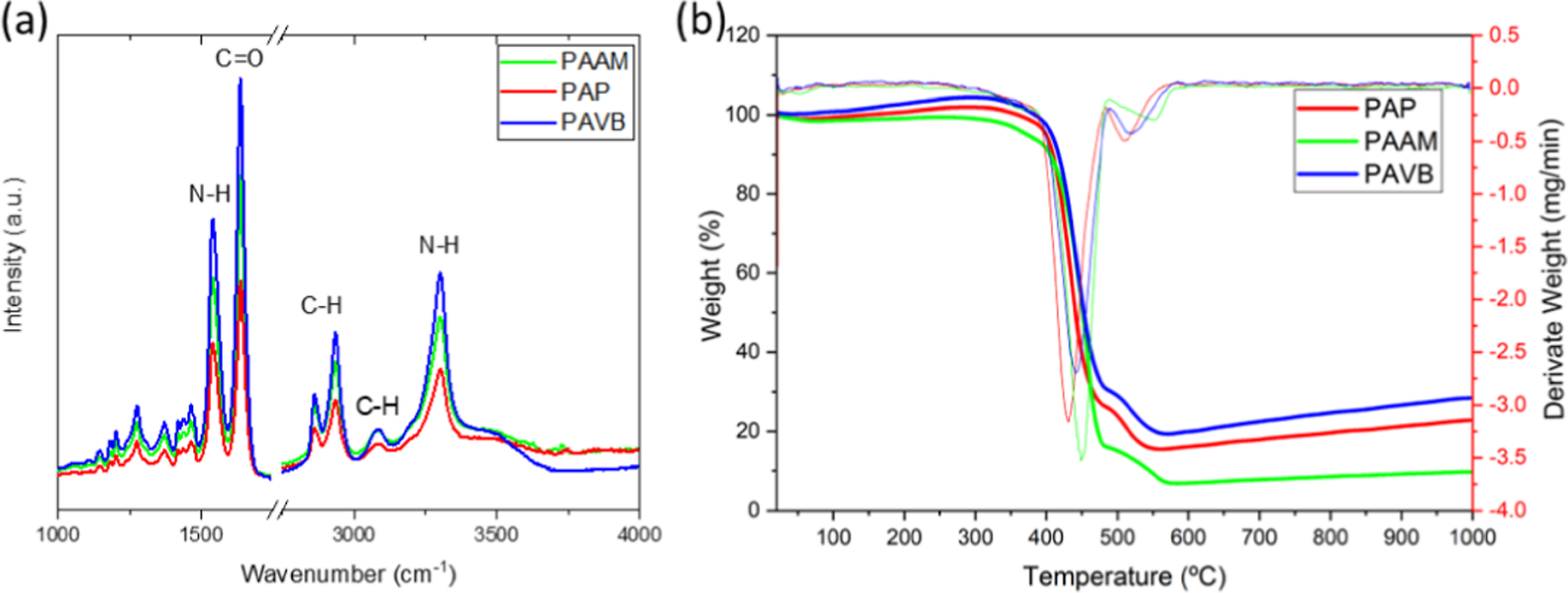
(a) ATR-FTIR spectroscopy of electrospun ultrathin fibers produced from PAAM (green), PAP (red) and PAVB (blue). (b) TGA of electrospun membranes produced from PAAM, PAP and PAVB.

The ATR-FTIR spectroscopy analysis (Figure 3a) provides detailed insights into the chemical structure of the PAP, PAVB, and PAAM fibers. The spectra reveal the presence of characteristic polyamide functional groups. Specifically, the band at 1541 cm^–1^ is attributed to NH angular deformation, while the band at 1637 cm^–1^ corresponds to the angular deformation of C=O. Additionally, the CH stretching is observed at 2933 cm^–1^, the CH stretch at 3082 cm^–1^, and the N–H stretch at 3301 cm^–1^.^41−43^ The intensity of peaks associated with polar groups, such as C=O, N–H, and O–H, is more pronounced in the PAVB fibers, indicating a higher concentration of these polar groups. This increased presence of polar groups enhances the hydrophilicity of the PAVB fibers, as evidenced by the stronger FTIR signals. In comparison, the PAP fibers exhibit lower peak intensities for these polar groups, reflecting a reduced concentration and consequently lower hydrophilicity.

The FTIR results align with the contact angle measurements discussed earlier, confirming the relative hydrophilicity of the different fiber groups. The PAVB fibers, with their higher concentration of polar groups, demonstrate the highest hydrophilicity. This is followed by the PAAM fibers, which show intermediate levels of polar groups and hydrophilicity. The PAP fibers, with the least concentration of polar groups, exhibit the lowest hydrophilicity. These findings are crucial for understanding the surface properties and potential applications of the fibers. The higher hydrophilicity of the PAVB fibers can be advantageous in applications requiring enhanced interaction with water or biological fluids. On the other hand, the lower hydrophilicity of the PAP fibers could be beneficial in applications where water repellency is desired, such as in protective face masks.

The ATR-FTIR spectroscopy analysis corroborates the contact angle measurements, providing a comprehensive understanding of the chemical structure and surface properties of the PAP, PAVB, and PAAM fibers. The higher intensity of polar group peaks in the PAVB fibers underscores their enhanced hydrophilicity, making them suitable for applications where this property is advantageous.

The thermal analysis (Figure 3b) of the PAP, PAVB, and PAAM fiber groups provides important insights into their thermal stability and decomposition behavior. The thermogravimetric analysis (TGA) curves reveal distinct phases of weight loss corresponding to different thermal events. The initial weight reduction observed up to 370 °C is attributed to the volatilization of both bound and unbound volatile constituents. This phase indicates the loss of moisture and other low-molecular-weight components that are either physically adsorbed or weakly bound within the fiber matrix.

As the temperature increases from approximately 370 °C to about 480 °C, the primary thermal breakdown of the polymer chains occurs. This phase corresponds to the thermal decomposition of the main structural components of the polyamide fibers, leading to significant weight loss. The decomposition process involves the breaking of polymeric bonds, resulting in the formation of smaller volatile compounds.^44^ Beyond 480 °C, the subsequent phase denotes the thermal degradation of the polymer backbones. This stage is characterized by the continued breakdown of the polymer structure, ultimately leading to the formation of ash residues. The presence of ash indicates the complete thermal degradation of the organic components, leaving behind inorganic residues.

The thermal stability of the fibers is a critical parameter for their application in environments where high temperatures may be encountered. The observed thermal behavior suggests that the PAP, PAVB, and PAAM fibers possess adequate thermal stability for most practical applications. The initial weight loss up to 370 °C and the primary decomposition phase up to 480 °C indicate that these materials can withstand significant thermal stress before undergoing major degradation. The thermal analysis of the PAP, PAVB, and PAAM fibers reveals their decomposition behavior and thermal stability. The initial volatilization of volatile constituents up to 370 °C, followed by the breakdown of polymer chains up to 480 °C, and the subsequent thermal degradation of polymer backbones, provide a comprehensive understanding of the thermal properties of these fibers.

**Figure 4:**
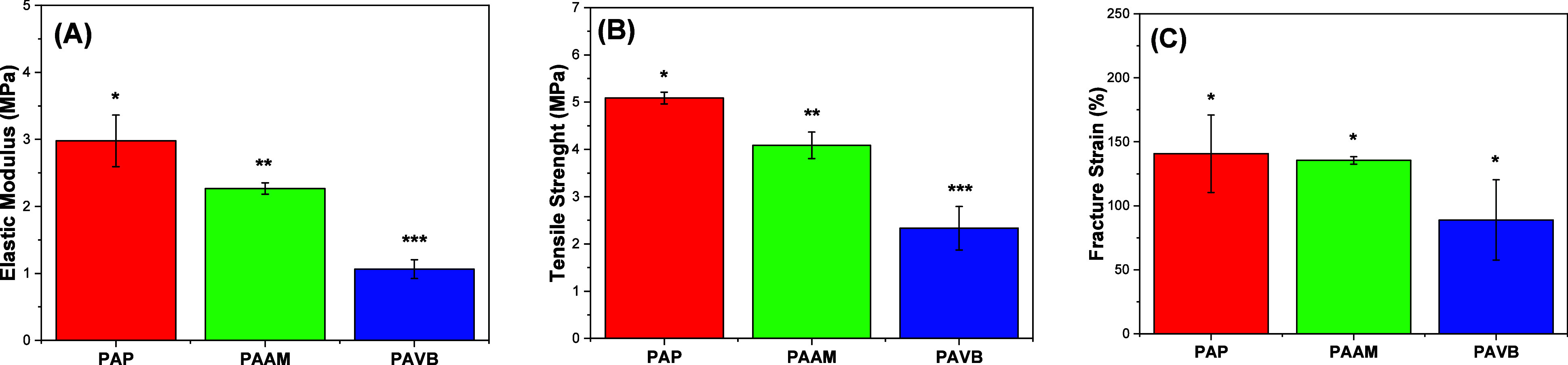
Mechanical properties of electrospun membranes. Measurement of (a) elastic modulus, (b) tensile strength and (c) fracture strain of electrospun ultrathin fibers. Data are exposed as mean ± standard error (*N* = 3). Columns with different symbols indicate a statistically significant difference between the analyzed groups. (*p < 0.5), (**p < 0.05) and (***p < 0.01).

The mechanical properties of the electrospun ultrathin fibers made from different polyamides were evaluated using tensile tests to determine their suitability for use as filter materials in face masks. The ability to withstand external loads is a crucial requirement for such applications. The tensile strength values observed were: 2.33 ± 0.46 MPa for PAVB, 4.08 ± 0.28 MPa for PAAM, and 5.09 ± 0.12 MPa for PAP (Figure 4b). These values indicate the maximum stress the fibers can endure before breaking, reflecting their mechanical robustness. Elongation at break, another important mechanical property, was also measured. The PAAM fibers exhibited an elongation at break of 2.96%, whereas the PAP fibers showed a significantly higher elongation at break of 140.72 ± 30.32% (Figure 4c). This substantial difference suggests that the PAP fibers are not only stronger but also more ductile, making them more capable of deforming under stress without breaking.

The mechanical properties measured in this study align with those reported in previous studies on similar ultrathin fiber characteristics.^45,46^ The PAP group demonstrated superior mechanical properties compared to the other groups, making them particularly suitable for use in face masks where both strength and flexibility are critical. The high tensile strength and elongation at break of the PAP fibers suggest that they can withstand mechanical stresses encountered during regular use without compromising their structural integrity. This resilience is essential for maintaining the protective efficacy of the mask over time. Although the PAVB and PAAM fibers exhibited lower tensile strength and elongation at break, they still possess adequate mechanical properties for use in filter applications.

The mechanical properties of the PAP, PAVB, and PAAM fibers confirm their viability as materials for producing filters and face masks. The PAP fibers, with their exceptional tensile strength and elongation at break, stand out as the most promising candidates. However, the PAVB and PAAM fibers also meet the necessary mechanical requirements, making all three groups suitable for such applications. This study highlights the potential of electrospun polyamide fibers in developing high-performance, durable, and flexible filter materials for face masks.

**Figure 5:**
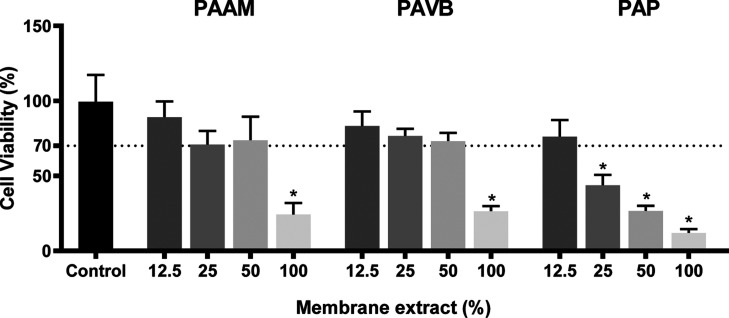
Percentage of cell viability. L929 cells were exposed to PAAM, PAVB and PAP extracts (12.5, 25, 50 and 100%) for 72 h.

The in vitro cytotoxicity of the electrospun ultrathin fibers from the PAP, PAVB, and PAAM groups was evaluated using the MTT viability assay on L929 cells. The results, shown in Figure 5, indicate that at a 100% concentration of the extract, all polymers exhibited a cytotoxic effect, reducing cell viability to approximately 70% (*p < 0.05). At lower extract concentrations, the cytotoxicity varied. Specifically, all extracts at a 12.5% concentration showed no cytotoxicity, maintaining high cell viability. Similarly, the 25% and 50% concentrations of PAAM and PAVB extracts did not induce significant cell death, suggesting their nontoxic nature at these intermediate concentrations.

The PAP fibers, identified as the most hydrophobic through contact angle testing, demonstrated limited interaction with the culture medium. This limited interaction likely contributes to their lower cytotoxicity at reduced concentrations, as hydrophobic fibers are less prone to releasing potentially harmful substances into the medium. Conversely, the PAVB fibers, which are more rigid and hydrophilic with a high elastic modulus (∼1.06 MPa), displayed a higher propensity to absorb water and interact positively with the cells. This interaction is beneficial in maintaining cell viability, as evidenced by the nontoxic response at 25% and 50% extract concentrations.

These findings underscore the importance of fiber hydrophobicity and mechanical properties in influencing cytotoxicity. The more hydrophilic and rigid PAVB fibers exhibit better biocompatibility at low concentrations, while the hydrophobic PAP fibers, despite being less interactive with the medium, also show nontoxic behavior at similar concentrations. The in vitro cytotoxicity tests indicate that the extracts from the electrospun ultrathin fibers are nontoxic at lower concentrations, making them suitable for biomedical applications such as filters in face masks.

**Figure 6:**
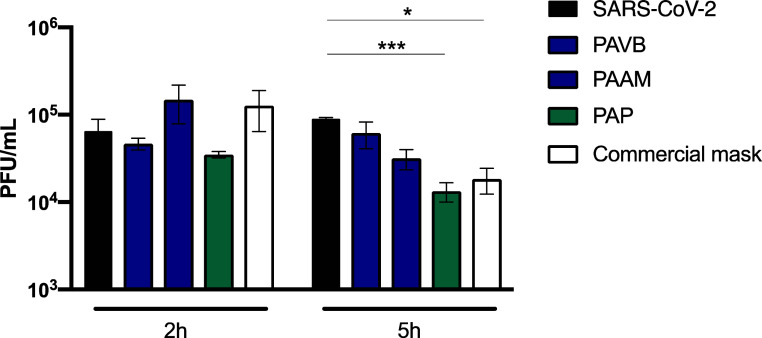
Antiviral activity against SARS-CoV-2. Contact of polyamide electrospun ultrathin fibers groups with SARS-CoV-2 (P.1 variant) for 2 and 5 h. Data are exposed as mean ± standard error. *p < 0.05, ***p < 0.01, one-way ANOVA (Dunnett’s posttest).

The antiviral activity of the electrospun ultrathin fibers from the PAP, PAVB, and PAAM groups was assessed against the SARS-CoV-2 P.1 variant, alongside commercial masks from the Brazilian textile industry (Figure 6). The evaluation was conducted at two different time points: 2 and 5 h after contact with the virus. Five hours postcontact, the PAP fibers and commercial masks demonstrated significantly increased virucidal activity. The PAP fibers showed a highly significant increase in virucidal activity (***P < 0.001), while the commercial masks also exhibited a notable increase (*P < 0.05) compared to the control and other groups. This indicates that the PAP fibers have a superior capacity to inactivate the virus over a longer duration of contact.

However, at the 2 h mark, none of the fiber groups, including PAP, PAVB, and PAAM, nor the commercial masks, displayed significant virucidal activity against the P.1 variant. This suggests that a longer interaction time is necessary for the antiviral properties of the PAP fibers to manifest effectively. The enhanced virucidal activity observed after 5 h could be attributed to the unique surface properties and the potential gradual release of antiviral agents from the fibers, which require a longer duration to interact effectively with the virus particles. The hydrophobic nature and high tensile strength of the PAP fibers, as discussed earlier, might also contribute to their prolonged and effective virucidal action.

Overall, these findings highlight the potential of PAP nanofibers as an alternative material for protective face masks, offering significant antiviral properties against SARS-CoV-2 with sufficient contact time. The prolonged virucidal activity of PAP fibers underscores their promise in developing advanced filtration systems capable of providing enhanced protection against viral transmission.

Table 1 presents a comparative analysis of various electrospun materials employed in air filtration, with an emphasis on their potential use in face masks. The table highlights crucial parameters such as fiber size distribution, contact angle measurements, tensile strength, cell viability, and antiviral activity, providing a comprehensive overview of the performance characteristics of each material.

**Table 1:** Comparative Analysis of Filtration Performance in Electrospun Air Filters

filters type	fiber size distribution	contact angle measurements	tensile strength	cell viability	antiviral activity against SARS-CoV-2 or bacterial activity for application to viruses	ref
Biodegradable and high-performance multiscale structured nanofiber membrane as mask filter media via poly(lactic acid) electrospinning	0.187 ± 0.013 μm	116.3 to 136.7°	Tensile strength of 10.52 MPa, Young’s modulus of 173 MPa		In this research, a novel approach is suggested for the production of PLA nanofibers. The authors suggest that these filters could serve as promising alternatives to current commercial masks in the future	(36)
PVDF/DBS/F-SiO2 electrospinning membrane		PVDF: 139.3°				(47)
		PVDF/DBS: 139.7°				
		PVDF/DBS/F-SiO_2_: 144.2°				
PEI/FAS/F-ZIF67 nanofibrous membranes	0.540 μm	130°				(48)
Antiviral nanofibers containing various Cu salts and ZnO nanorods by electrospinning	CuBr10/ZnO24: 0.535 ± 0.870				The findings suggest that ZnO nanorods inhibit the oxidation and degradation of the antiviral activity of Cu(I), leading to effective virus inactivation even in the absence of light	(10)
	CuBr17/ZnO17: 0.636 ± 0.1699					
	CuBr24/ZnO10: 0,6729 ± 0,1246 μm					
Needleless electrospun phytochemicals encapsulated nanofibre based 3-ply biodegradable mask for combating COVID-19 pandemic	8 ± 0.2 μm	143°			Bacterial efficiency	(49)
This work	PAAM = 0.74 μm	PAAM = 120.7°	PAVB = 2.33 ± 0.46 MPa	The 25% concentration of PAAM, PAVB and PAP extracts did not promoted cell death, indicating their nontoxic activity in normal cells	Good potential of ultrathin fibers to be used as an alternative material against SARS-CoV-2	
	PAP = 0.59 μm	PAP = 130°	PAAM = 4.08 ± 0.28 MPa			
	PAVB = 0.69 μm	PAVB = 89.1°	PAP = 5.09 ± 0.12 MPa			

The filtration performance of these materials is predominantly influenced by their fiber size distribution and morphology. Smaller and more uniform fibers, such as those of PAP with a diameter of 0.59 μm, enhance the filtration efficiency by providing a larger surface area for capturing particles and pathogens. This uniformity is critical as it minimizes variations in pore size, ensuring consistent performance and eliminating weak points that could compromise the filter’s efficacy.

Contact angle measurements provide insight into the hydrophobicity of the materials, which is a vital factor in determining the interaction between the fibers and pathogens. For instance, the PAP fibers exhibit a contact angle of 130°, indicating a significant degree of hydrophobicity. This property is advantageous as it can repel waterborne pathogens and prevent the absorption of moisture, which is particularly beneficial in a face mask application where maintaining dryness is crucial for comfort and effectiveness.

Mechanical properties, such as tensile strength, are also essential for evaluating the durability and resilience of the filters. The PAP fibers demonstrate the highest tensile strength of 5.09 ± 0.12 MPa, suggesting they can withstand mechanical stresses during use without degrading, thus maintaining their filtration performance over time.

Cell viability tests indicate the biocompatibility of the materials. The PAP fibers show no cytotoxicity at lower concentrations, making them safe for prolonged contact with human skin. This characteristic is particularly important for face masks, as materials that cause irritation or adverse reactions would not be suitable for such applications.

**Figure 7:**
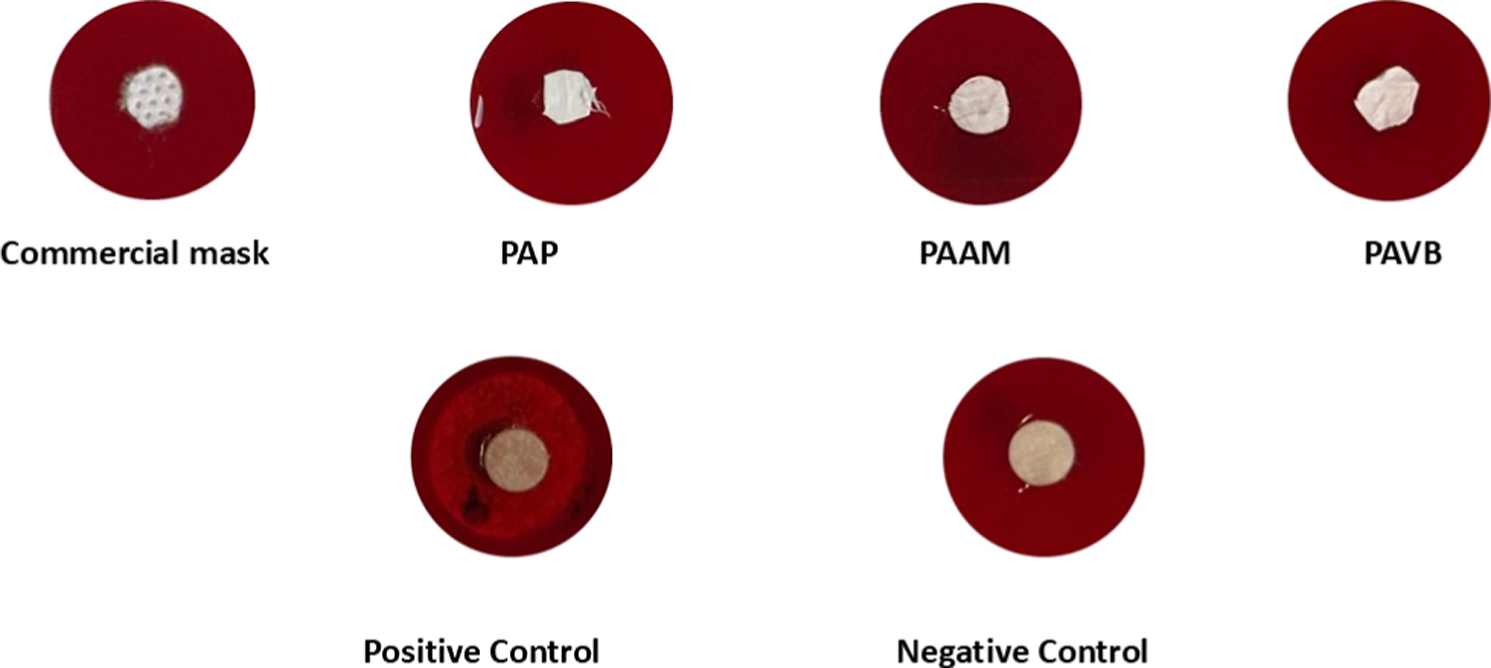
Hemolytic characteristic: hemolysis on blood agar plates for the groups of commercial masks, PAP, PAAM, PAVB, positive control (hemolytic) and negative control (nonhemolytic) for 24 h.

The hemolytic activity analysis displayed in Figure 7 offers valuable insights into the interaction of the ultrathin fibers with biological systems, particularly in terms of blood compatibility. The PAP, PAAM, and PAVB fibers demonstrated a nonhemolytic profile, as no significant hemolysis zones were observed around the discs in sheep blood agar. This nonhemolytic behavior is essential for ensuring that the fibers are suitable for applications in which direct or indirect contact with blood may occur, such as in respiratory face masks where accidental exposure to blood is possible.

These results align with the previously assessed biocompatibility of the fibers, as indicated by their noncytotoxicity at lower concentrations in cell viability tests. The absence of hemolytic activity, alongside the hydrophobic nature observed in PAP and PAAM fibers (contact angles of 131 and 120.7°, respectively), further underscores their suitability for biomedical applications. The combination of noncytotoxicity, hydrophobicity, and nonhemolytic properties makes the PAP fibers particularly promising for prolonged human use. Additionally, the enhanced mechanical robustness of PAP, as demonstrated by its tensile strength and elongation at break, supports its potential for reliable and durable protective equipment.

Together, the hemolytic and cell viability results suggest that PAP fibers offer an optimal balance of biocompatibility, mechanical properties, and safety, making them well-suited for developing advanced air filtration and protective face masks with low risk of adverse biological interactions.

Lastly, the table highlights the antiviral activity against SARS-CoV-2. The PAP fibers exhibit significant virucidal activity, which is attributed to their smaller fiber size and increased hydrophobicity. This suggests that PAP nanofibers could be an effective material in face masks, offering both mechanical filtration and antiviral properties.

Overall, the data in Table 1 underscore the multifaceted criteria that must be considered when developing and selecting materials for air filtration and face masks. The PAP fibers, with their superior mechanical properties, biocompatibility, and antiviral activity, emerge as a promising candidate for high-performance filtration applications.

Thus, Table 1 lists a comparison between several electrospun materials, from different works, for application in filters. Obviously, in terms of effective studies with viruses, this study is the only one to carry out tests with the virus, the other works carry out tests with bacteria and the material’s capacity as a filter. In addition, cytotoxicity tests are carried out and thus suggest an ideal amount to be used. In other studies, close fiber sizes are observed, especially when dealing with polyamide fibers.

## 4 Conclusions

This study demonstrates the potential of electrospun polyamide ultrathin fibers as effective materials for personal protective equipment (PPE) applications, particularly in face masks aimed at antiviral protection. The produced fibers, with diameters ranging from 0.59 to 0.74 μm, exhibit regular morphology, porosity, and distinct hydrophobic properties in the PAP and PAAM groups, making them suitable for filtration applications where repelling moisture is critical. The chemical structure, as revealed by FTIR analysis, suggests that functional group distribution may contribute to the selective hydrophobic and hydrophilic properties observed in these materials. The mechanical properties of the fibers further reinforce their viability for PPE use, as the PAP fibers displayed superior tensile strength and flexibility, which are essential for withstanding prolonged wear and physical stress. In biocompatibility assessments, the PAP, PAAM, and PAVB groups maintained noncytotoxic profiles at lower concentrations, indicating their safety for contact with human skin. Furthermore, the hemolytic activity analysis confirmed the blood compatibility of these materials, with no hemolysis observed in any fiber group, a critical factor for biomedical applications. Notably, the PAP fibers exhibited enhanced virucidal activity against the SARS-CoV-2 Gamma variant (P.1), demonstrating significant viral inactivation after prolonged contact. This property, combined with nonhemolytic behavior and the absence of cytotoxicity, highlights the PAP fibers as a particularly promising candidate for face masks designed to provide both mechanical and biological protection. These findings emphasize the multifaceted potential of electrospun polyamide fibers, particularly PAP, in developing next-generation PPE that balances durability, biocompatibility, and antiviral efficacy. Further studies could explore surface modifications to optimize fiber properties for specific applications, contributing to advancements in protective barrier technologies.
